# Small duct and large duct type intrahepatic cholangiocarcinoma reveal distinct patterns of immune signatures

**DOI:** 10.1007/s00432-024-05888-y

**Published:** 2024-07-22

**Authors:** Simon Bernatz, Falko Schulze, Julia Bein, Katrin Bankov, Scherwin Mahmoudi, Leon D. Grünewald, Vitali Koch, Angelika Stehle, Andreas A. Schnitzbauer, Dirk Walter, Fabian Finkelmeier, Stefan Zeuzem, Thomas J. Vogl, Peter J. Wild, Maximilian N. Kinzler

**Affiliations:** 1 Department of Diagnostic and Interventional Radiology, University Hospital, Goethe University Frankfurt, Theodor-Stern-Kai 7, 60590 Frankfurt am Main, Germany; 2 Dr. Senckenberg Institute for Pathology, University Hospital, Goethe University Frankfurt, Theodor-Stern-Kai 7, 60590 Frankfurt am Main, Germany; 3 Frankfurt Cancer Institute (FCI), Goethe University Frankfurt, Theodor-Stern-Kai 7, 60590 Frankfurt am Main, Germany; 4 University Cancer Center Frankfurt (UCT), University Hospital, Goethe University Frankfurt, Theodor-Stern-Kai 7, 60590 Frankfurt am Main, Germany; 5 Department of Pediatric Oncology and Hematology, Charité, Universitätsmedizin Berlin, Corporate Member of Freie Universität Berlin and Humboldt-Universität zu Berlin, Augustenburger Platz 1, 13353 Berlin, Germany; 6 Medical Clinic 1, University Hospital, Goethe University Frankfurt, Theodor-Stern-Kai 7, 60590 Frankfurt am Main, Germany; 7 Department of General, Visceral, Transplant and Thoracic Surgery, University Hospital, Goethe University Frankfurt, Theodor-Stern-Kai 7, 60590 Frankfurt am Main, Germany; 8 Frankfurt Institute for Advanced Studies (FIAS), Frankfurt am Main, Germany

**Keywords:** Biomarker, Cholangiocarcinoma, Intrahepatic, Immune profiling, Pathology, molecular, Surgical oncology

## Abstract

**Purpose:**

Dedicated gene signatures in small (SD-iCCA) and large (LD-iCCA) duct type intrahepatic cholangiocarcinoma remain unknown. We performed immune profiling in SD- and LD-iCCA to identify novel biomarker candidates for personalized medicine.

**Methods:**

Retrospectively, 19 iCCA patients with either SD-iCCA (n = 10, median age, 63.1 years (45–86); men, 4) or LD-iCCA (n = 9, median age, 69.7 years (62–85); men, 5)) were included. All patients were diagnosed and histologically confirmed between 04/2009 and 01/2021. Tumor tissue samples were processed for differential expression profiling using NanoString nCounter® PanCancer Immune Profiling Panel.

**Results:**

With the exception of complement signatures, immune-related pathways were broadly downregulated in SD-iCCA vs. LD-iCCA. A total of 20 immune-related genes were strongly downregulated in SD-iCCA with DMBT1 (log2fc = -5.39, *p* = 0.01) and CEACAM6 (log2fc = -6.38, *p* = 0.01) showing the strongest downregulation. Among 7 strongly (log2fc > 2, *p* ≤ 0.02) upregulated genes, CRP (log2fc = 5.06, *p* = 0.02) ranked first, and four others were associated with complement (C5, C4BPA, C8A, C8B). Total tumor-infiltrating lymphocytes (TIL) signature was decreased in SD-iCCA with elevated ratios of exhausted-CD8/TILs, NK/TILs, and cytotoxic cells/TILs while having decreased ratios of B-cells/TILs, mast cells/TILs and dendritic cells/TILs. The immune profiling signatures in SD-iCCA revealed downregulation in chemokine signaling pathways inclulding JAK2/3 and ERK1/2 as well as nearly all cytokine-cytokine receptor interaction pathways with the exception of the CXCL1/CXCR1-axis.

**Conclusion:**

Immune patterns differed in SD-iCCA versus LD-iCCA. We identified potential biomarker candidate genes, including CRP, CEACAM6, DMBT1, and various complement factors that could be explored for augmented diagnostics and treatment decision-making.

**Supplementary Information:**

The online version contains supplementary material available at 10.1007/s00432-024-05888-y.

## Introduction

Cholangiocarcinoma (CCA) is a highly heterogeneous malignancy originating from the intrahepatic biliary epithelium (iCCA) or from extrahepatic bile ducts (eCCA). Patients with CCA have a poor prognosis and the incidence of iCCA is rising globally, accounting for about 10–15% of primary liver cancers (Bertuccio et al. 2013; Bridgewater et al. 2014). With surgical resection being the sole curative treatment option, the prognosis for iCCA patients remains unfavorable (Groot Koerkamp and Fong 2014). The new standard of care in the palliative setting is the combination of chemotherapy with gemcitabine and cisplatin and the immune checkpoint inhibitors durvalumab or pembrolizumab, leading to a median overall survival of 12.8 and 12.7 months, respectively (Oh et al. 2022; Kelley et al. 2023).

In recent years, histopathological characterization of iCCA revealed two distinct subtypes according to the size of the affected bile duct, which led to implementation in the WHO classification (WHO Classification of Tumours 2019, 5th ed. Vol. 1. Digestive System Tumours, 2019. [Online]. Available: https://publications.iarc.fr/ Book-And-Report-Series/ Who-Classification-Of- Tumours/Digestive-System-Tumours-2019.'; Kendall et al. 2019; Aishima and Oda 2015). On the one hand, small duct type iCCA (SD-iCCA) was found to be more peripheral in the liver and resembling a ductular and cholangiolar type (Liau et al. 2014; Chung and Park 2022). On the other hand, the large duct type (LD-iCCA) arises from large intrahepatic ducts closer to the liver hilum and contains mainly mucin-producing columnar tumor cells (Hayashi et al. 2016; Sigel et al. 2018; Chung and Park 2022). Remarkably, both subtypes differ in underlying diseases, survival, response to chemotherapy, and molecular alterations, emphasizing clinically relevant subtype heterogeneity (Kinzler et al. 2022; Aishima and Oda 2015; Chung et al. 2020; Kendall et al. 2019; Gerber et al. 2022).

Immunotherapy emerged in the last decade and revolutionized treatments and outcomes across multiple cancer entities, including CCA (Pan et al. 2020; Greten et al. 2023; Fiste et al. 2021). Although the results of the TOPAZ-1 and KEYNOTE-966 trial have opened new perspectives for palliative CCA patients (Oh et al. 2022; Kelley et al. 2023), immunotherapeutic approaches in the management of iCCA patients remain challenging as the immunosuppressive tumor microenvironment (TME) plays a pivotal role in iCCA progression and, thereby, potential response to immunotherapeutic agents (Greten et al. 2023; Banales et al. 2020). The highly reactive TME comprises a variety of immune cells, including cancer-associated fibroblasts, tumor-associated macrophages*,* endothelial cells, and lymphocytes (Xia et al. 2022b; Job et al. 2020; Banales et al. 2020), but detailed characteristics are lacking. Here, improved characterization of the immune landscape in iCCA holds substantial clinical potential, both for predicting response to immunotherapy and for identifying novel treatment strategies. A few preliminary studies investigated immune signatures as predictive biomarkers in iCCA (Xia et al. 2022b; Yoon et al. 2021; Konishi et al. 2022; Yugawa et al. 2021; Jing et al. 2019). However, differences in immune signatures between SD- and LD-iCCA remain unknown, and exploratory studies are lacking so far.

We hypothesized that the heterogeneity of iCCA subtypes is reflected in their immune patterns and that these differences could hold a significant potential for diagnostic and therapeutic personalized medicine.

## Materials and methods

### Patient cohort

All patients treated with surgically resected (R0, R1) intrahepatic cholangiocarcinoma at Frankfurt University Hospital between December 2005 and December 2021 were retrospectively screened. Clinical data (sex, date of birth, tumor stage, tumor size, laboratory parameters, and comorbidities) were collected from electronic medical records. iCCA were staged according to the 8th edition of the classification of the Union for International Cancer Control (UICC). Tissue samples used in this study were provided by the University Cancer Center Frankfurt (UCT). Written informed consent was obtained from all patients at the time of initial surgery, or written informed consent was waived if the patient was deceased. The study was approved by the institutional Review Boards of the UCT and the Ethical Committee at the University Hospital Frankfurt (project-number: SGI-1-2021, SGI-3-2021).

### Study design

**Fig. 1:**
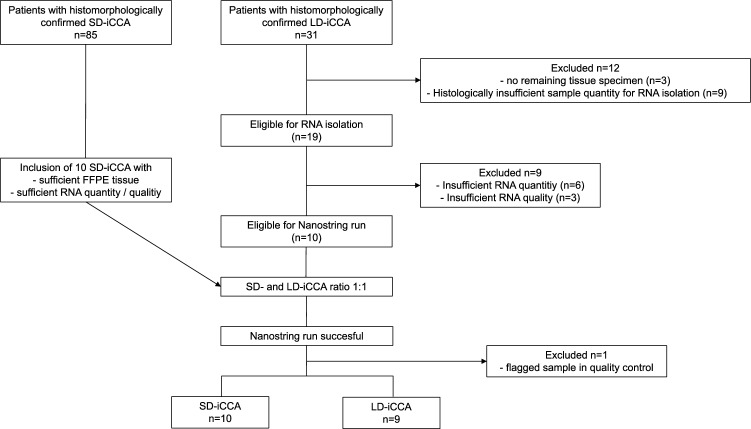
STARD Flowchart of patient inclusion into the study. *STARD* Standards for Reporting Diagnostic Accuracy Studies

Hematoxylin and eosin (HE) slides and formalin-fixed paraffin-embedded (FFPE) tissue were retrieved from the archive of the Dr. Senckenberg Institute of Pathology, University Hospital Frankfurt. According to the WHO Classification of Tumours, Digestive System Tumours, 5th edition, Volume 1 ('WHO Classification of Tumours 2019, 5th ed. Vol. 1. Digestive System Tumours, 2019. [Online]. Available: https://publications.iarc.fr/ Book-And-Report-Series/ Who-Classification-Of- Tumours/Digestive-System-Tumours-2019.'), all samples examined in the present study were recently analyzed both histomorphologically and immunohistochemically by an expert hepatobiliary pathologist and assigned to the respective subtype in our previously published work (Kinzler et al. 2022). Retrospectively, 20 iCCA-patients (n = 10, SD-iCCA and n = 10, LD-iCCA) who were diagnosed between 04/2009 and 01/2021, were included in the present study. Inclusion criteria: (1) Histologically confirmed treatment-naïve SD-iCCA or LD-iCCA. Exclusion criteria: (1) insufficient tissue sample / RNA -/ or NanoString® quality or quantity. To gain equal distribution of SD- and LD-iCCA in our study, the number of included SD-iCCA samples was adjusted to the less frequent LD-iCCA type. Figure 1 depicts the flowchart of patient inclusion according to Standards for Reporting Diagnostic Accuracy Studies (STARD).

### Ribonucleic acid (RNA) isolation and immune profiling analysis

Representative tumor material was punched out of FFPE blocks using a 1 mm core needle. RNA was isolated using the truXTRAC FFPE total NA Kit (Covaris, Woburn, MA, USA) based on focused ultrasonification and column purification according to the manufacturer's instructions. NanoString nCounter® Platform and PanCancer Immune Profiling Panel were used to enrich a commercially available function specific panel of 770 genes by hybrid capture technique (NanoString®, Seattle, WA, USA) as previously published (Kinzler et al. 2023). NanoString nSolver™ software v4 and implemented nCounter® Advanced Analysis module v2.0.134 were used for subsequent raw data processing and normalization by internal controls following differential supervised analysis of SD-iCCA (n = 10) versus baseline of LD-iCCA (n = 9). Quality control was done with default settings as previously described (Preusse et al. 2021). One sample (LD-iCCA) was flagged in quality control as the percentage of successfully scanned fields of view in its cartridge lane was below the threshold of 75%, and we excluded this sample for further analysis. Gene expression of LD-iCCA was set as baseline for the comparative analysis. For pathway analysis of differentially expressed genes, Enrichr (Chen et al. 2013; Kuleshov et al. 2016) was used for functional enrichment analysis for Gene Ontology to identify gene-sets for biological processes. For further differential expression analysis, we used the following cut-offs: log2 fold change < -2 or > 2 and *p*-Value ≤ 0.05 after Benjamini-Hochberg (BH) correction. T-distributed stochastic neighbor embedding (t-SNE) analysis and plots were performed in Python 3.7.6.

### Statistical analysis

We compared baseline clinicopathological characteristics between patients with SD- and LD-iCCA. For statistical analysis, two-sided Students t-test was used for continuous variables and Likelihood Ratio for nominal / ordinal data.

## Results

### Study population

In total, 19 patients with iCCA were included in this study. Ten patients with SD-iCCA (median age, 63.1 years (45–86); men, 4) and nine patients with LD-iCCA (median age, 69.7 years (62–85); men, 5) were analyzed. The groups did not differ in clinicopathological characteristics including tumor size, the occurrence of multiple tumors, UICC stadium, performance status or selected laboratory values like CA-19/9, bilirubin, or lactate dehydrogenase (Table 1). Interestingly, patients with LD-iCCA were more likely to have hepatolithiasis (*p* = 0.049). However, patients with SD- and LD-iCCA did not differ in any other common risk factors including viral hepatitis, primary sclerosing cholangitis or liver cirrhosis. Further clinical characteristics are depicted in Table 1.

**Table 1:** Clinical and epidemiological characteristics

Characteristics	SD-iCCA (n = 10), No. (%)	LD-iCCA (n = 9), No. (%)	*p*-value
Sex			0.525
Female	6 (60)	4 (44.4)	
Male	4 (40)	5 (55.6)	
Age at initial diagnosis			0.172
Mean, years, (range)	63.1 (45–86)	69.7 (62–85)	
UICC			0.695
1a	1 (10)	2 (22.2)	
1b	3 (30)	2 (22.2)	
2	3 (30)	1 (11.1)	
3a	1 (10)	0 (0)	
3b	2 (20)	3 (33.3)	
4	0 (0)	1 (11.1)	
ECOG			0.72
0	7 (70)	7 (77.8)	
1	3 (30)	2 (22.2)	
CA-19/9 (ng/ml)			1
< 37	5 (50)	4 (44.4)	
≥ 37	5 (50)	4 (44.4)	
n.a.	0 (0)	1 (11.1)	
Tumor size (cm)			0.541
≤ 5	3 (30)	4 (44.4)	
> 5	7 (70)	5 (55.6)	
Single Tumor			0.106
Yes	4 (40)	7 (77.8)	
No	6 (60)	2 (22.2)	
Pathological grade			0.912
Grade 2	8 (80)	7 (77.8)	
Grade 3	2 (20)	2 (22.2)	
R status			0.121
R0	8 (80)	4 (44.4)	
R1	2 (20)	5 (55.6)	
L status			0.183
L0	9 (90)	5 (55.6)	
L1	1 (10)	3 (33.3)	
Lx	0 (0)	1 (11.1)	
Pn status			0.104
Pn0	7 (70)	3 (33.3)	
Pn1	2 (20)	5 (55.6)	
Pnx	1 (10)	1 (11.1)	
Recurrence			0.285
Yes	3 (30)	5 (55.6)	
No	7 (70)	4 (44.4)	
Hepatolithiasis			0.049
Yes	0 (0)	3 (33.3)	
No	10 (100)	6 (66.7)	
Viral hepatitis			0.305
Yes	0 (0)	1 (11.1)	
No	10 (100)	8 (88.9)	
PSC			0.357
Yes	1 (10)	0 (0)	
No	9 (90)	9 (100)	
Diabetes			0.884
Yes	3 (30)	3 (33.3)	
No	7 (70)	6 (66.7)	
Liver cirrhosis			0.305
Yes	0 (0)	1 (11.1)	
No	10 (100)	8 (88.9)	
LDH			
< 248	5 (50)	4 (44.4)	0.953
≥ 248	4 (40)	3 (33.3)	
n.a.	1 (10)	2 (22.2)	
Bilirubin			0.063
< 1.4	9 (90)	6 (66.7)	
≥ 1.4	0 (0)	3 (33.3)	
n.a.	1 (10)	0 (0)	

For statistical analysis, two-sided Students *t*-test was used for continuous variables and Likelihood Ratio for nominal / ordinal data. Data is shown as absolute numbers (%) or median (min–max)

*CA-19/9* carbohydrate antigen 19-9, *ECOG* Eastern Cooperative Oncology Group, *LDH* lactate dehydrogenase, *LD-iCCA* large duct type intrahepatic cholangiocarcinoma, *n.a.* not available, *UICC* Union for International Cancer Control, *SD-iCCA* small duct type intrahepatic cholangiocarcinoma

### Immune-pathway scores were downregulated in SD-iCCA with the exception of complement signatures

**Fig. 2:**
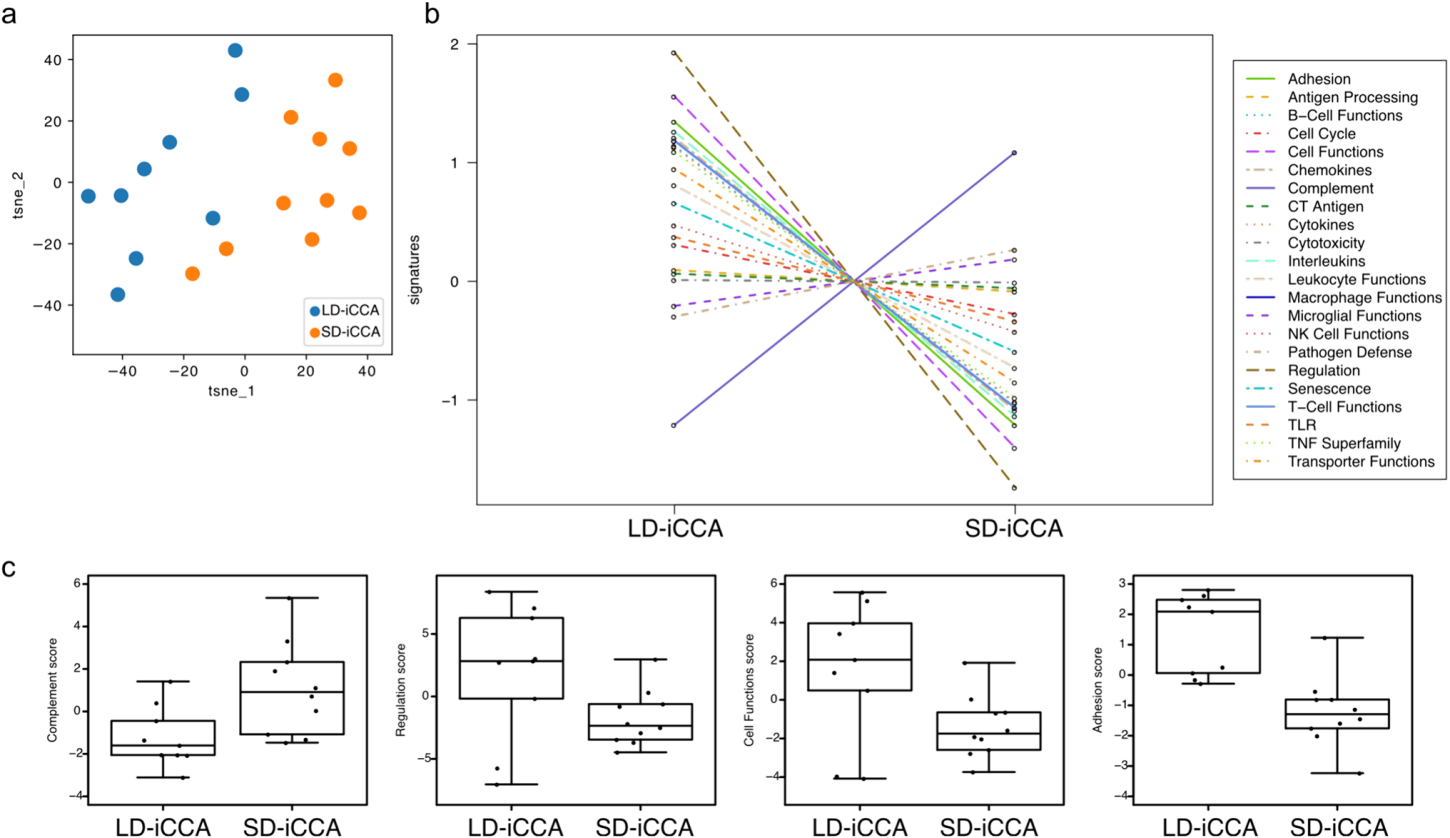
Dominant downregulation in immune pathway scores in small-duct type intrahepatic cholangiocarcinoma**.** **a** T-SNE plot of comprehensive log2 normalized mRNA patient data, **b** trend plot of pathway signatures using NanoString® pathway score analysis tool and **c** boxplots of the top 4 differentially expressed pathway score signatures

To explore if patients with SD- and LD-iCCA differed in their immune cell signatures, we first performed unsupervised T-SNE analysis and found a clear separation into two groups (Fig. 2a). Next, we used pathway score analyses of the functionally annotated genes to explore distinct patterns. Here we found that the majority of pathways in patients with SD-iCCA were downregulated, especially pathways associated with regulation, cell function and adhesion, with the exception of complement signatures that were upregulated (Fig. 2b,c).

### SD- and LD-iCCA revealed strongly differentially regulated candidate genes

**Fig. 3:**
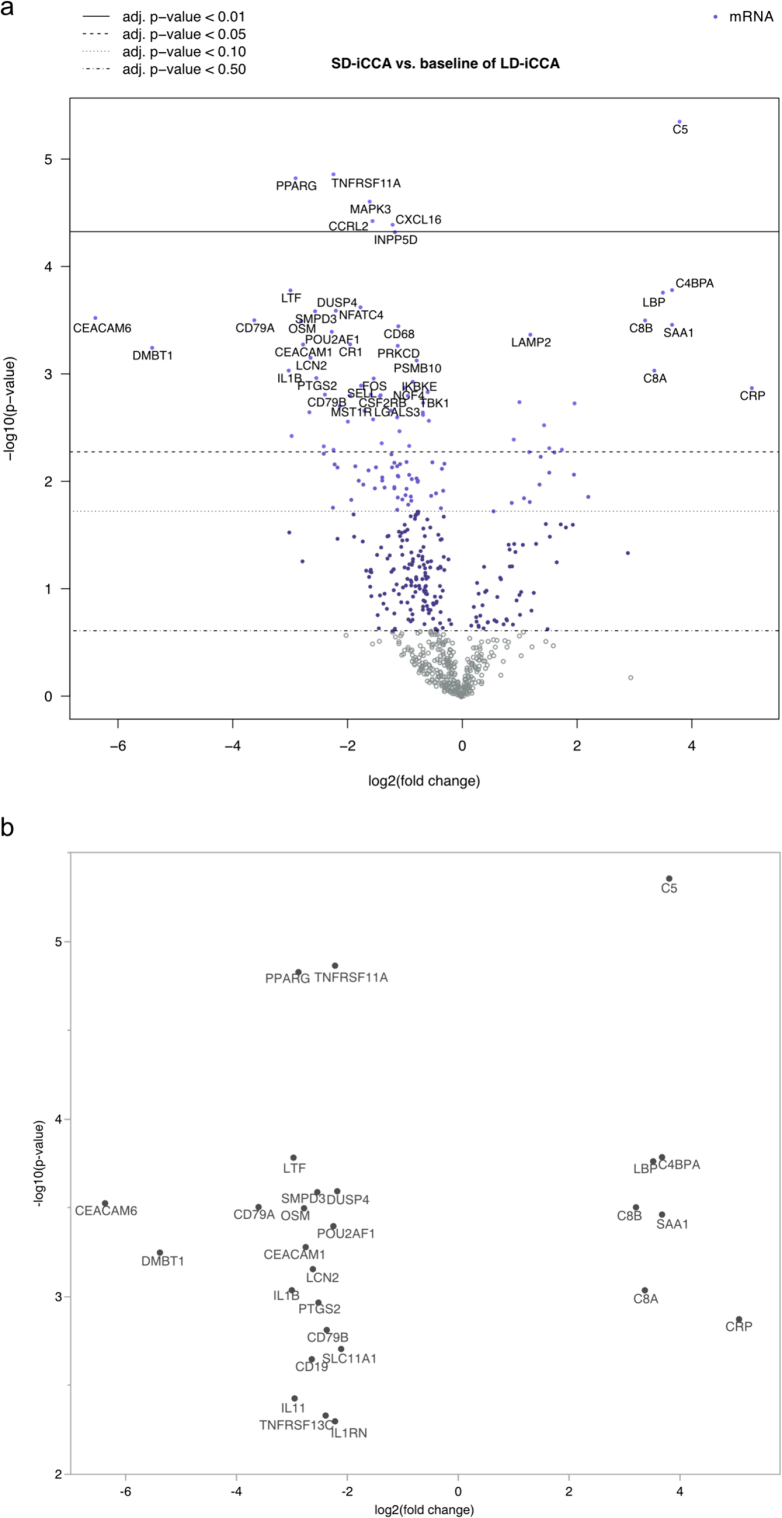
Differentially expressed genes between intrahepatic cholangiocarcinoma subtypes. **a** Volcano plot of all differentially expressed genes and only **b** strong and significantly (log2fc < -2 or > 2 and *p*-value < 0.05 with Benjamini–Hochberg correction)

Small- and large-duct type iCCA showed strong differences in the expression of immune-related genes (Fig. 3a). In total, 27 genes were strongly differentially expressed (log2fc > 2 or log2fc < -2, BH-*p* < 0.05) as depicted in Fig. 3b. CRP showed the strongest upregulation (log2fc = 5.06, *p* = 0.02, 95% CI [2.47–7.66]) in SD-iCCA. 57% (4/7) of the 7 upregulated genes in SD-iCCA were associated with complement, namely C5 (log2fc = 3.80, *p* = 0.003, 95% CI [2.67–4.93]), C4BPA (log2fc = 3.67, *p* = 0.01, 95% CI [2.18–5.17]), C8A (log2fc = 3.36, *p* = 0.02, 95% CI [1.71–5.00]), C8B (log2fc = 3.2, *p* = 0.01, 95% CI [1.81–4.59]). Most genes were strongly downregulated in SD-iCCA versus LD-iCCA, and the strongest downregulation was seen in carcinoembryonic antigen-related cellular adhesion molecule 6 (CEACAM6) (log2fc = – 6.38, *p* = 0.01, 95% CI [– 9.14 to – 3.62]), deleted in malignant brain tumor 1 (DMBT1) (log2fc = – 5.39, *p* = 0.01, 95% CI [– 7.88 to – 2.89]) and CD79A (log2fc = – 3.61, *p* = 0.01, 95% CI [– 5.18 to – 2.04]). See Table 2.

**Table 2:** Strongly differentially expressed genes in SD-iCCA vs. LD-iCCA

	Log2fc	95% CI	*p*-value	BH *p*-value	Gene sets
CRP	5.06	2.47/7.66	0.001	0.024	Transporter functions
C5	3.80	2.67/4.93	< 0.001	0.003	Complement
C4BPA	3.67	2.18/5.17	< 0.001	0.011	Complement
SAA1	3.67	2.06/5.28	< 0.001	0.012	
LBP	3.51	2.07/4.95	< 0.001	0.011	Macrophage functions
C8A	3.36	1.71/5.00	0.001	0.020	Complement
C8B	3.20	1.81/4.59	< 0.001	0.012	Complement
SLC11A1	– 2.12	– 3.26/– 0.98	0.002	0.027	Macrophage functions
DUSP4	– 2.19	– 3.12/– 1.26	< 0.001	0.012	
TNFRSF11A	– 2.23	– 2.95/– 1.50	< 0.001	0.003	TNF superfamily
IL1RN	– 2.23	– 3.58/– 0.872	0.005	0.048	Cytokines, Interleukins
POU2AF1	– 2.26	– 3.25/– 1.26	< 0.001	0.013	
CD79B	– 2.38	– 3.62/– 1.14	0.002	0.024	B-cell functions
TNFRSF13C	– 2.40	– 3.82/– 0.966	0.005	0.047	Regulation, TNF superfamily
PTGS2	– 2.53	– 3.79/– 1.27	0.001	0.022	Cytokines
SMPD3	– 2.55	– 3.62/– 1.48	< 0.001	0.012	Cell functions
LCN2	– 2.63	– 3.88/– 1.38	0.001	0.017	
CD19	– 2.65	– 4.08/– 1.22	0.002	0.028	B-cell functions, Regulation
CEACAM1	– 2.76	– 4.02/– 1.49	0.001	0.014	Adhesion
OSM	– 2.79	– 4.01/– 1.58	< 0.001	0.012	Cell functions
PPARG	– 2.89	– 3.84/– 1.94	< 0.001	0.003	
IL11	– 2.96	– 4.7/– 1.23	0.004	0.040	B-cell functions, cytokines, interleukins, T-cell functions
LTF	– 2.98	– 4.19/– 1.76	< 0.001	0.011	
IL1B	– 3.01	– 4.49/– 1.54	0.001	0.020	Chemokines, Cytokines, Interleukins, Pathogen Defense, Regulation
CD79A	– 3.61	– 5.18/– 2.04	< 0.001	0.012	
DMBT1	– 5.39	– 7.88/– 2.89	0.001	0.014	
CEACAM6	-6.38	-9.14 / -3.62	< 0.001	0.012	Adhesion

'Estimated log fold-change' estimates a gene's differential expression. For each gene, a single linear regression was fit with all selected covariates for prediction of expression to eliminate measured confounding and isolate the independent associations. The log2 fold change is presented, along with a *p*-value, an adjusted *p*-value or FDR (BH correction) and the 95% CI

*BH* Benjamini–Hochberg, *FDR* false discovery rate

### Cell type profiling signatures revealed two immune type subsets

**Fig. 4:**
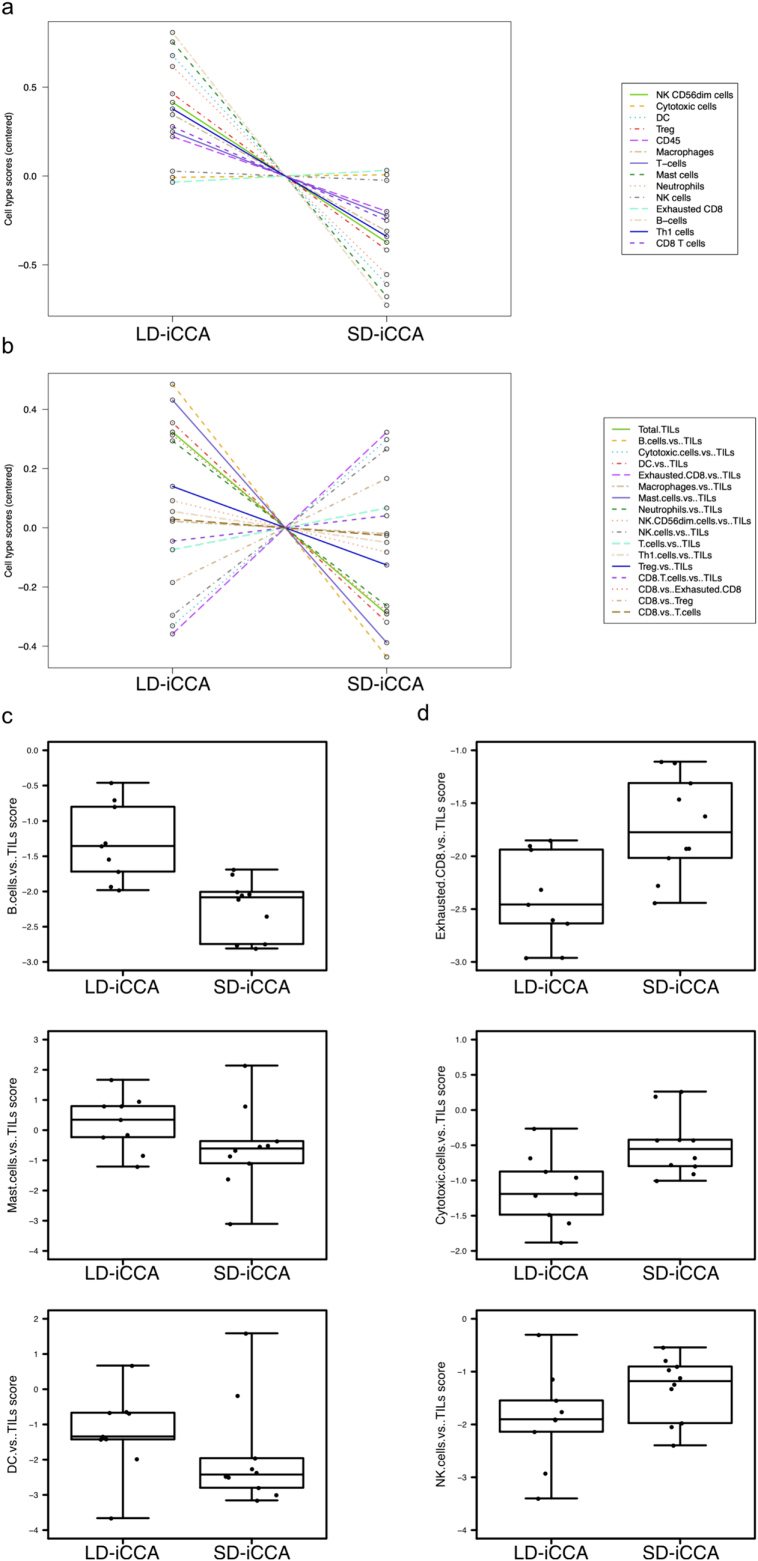
Cell type profiling revealed decreased immune infiltrates in SD-iCCA**.** Cell population abundance was measured based on characteristically expressed genes. The cell type abundance measurements are plotted against the tumor subtypes. **a** Total cell type scores and **b** the relative cell type scores. **c**, **d** Top differentially expressed cell type scores relative to total TILs **c** with downregulation in small-duct type iCCA or **d** upregulation in SD-iCCA

Total TILs and the absolute amount of the vast majority of cell type profiles were reduced in SD-iCCA (Fig. 4a). The relative cell type to TIL profile ratios revealed the most prominent decreases in B cells/TILs, mast cells/TILs and dendritic cells(DC)/TILs (Fig. 4b,c). Increased relative cell type profile ratios were revealed for exhausted CD8/TILs, cytotoxic cells/TILs and NK cells/TILs (Fig. 4b, d).

### Gene ontology term enrichment analysis

Applying Enrichr gene ontology term enrichment on the whole differentially expressed dataset, the biological processes were mostly related to a downregulation in the subtotal cytokine-cytokine receptor interaction signatures (Suppl. Fig. 1). Only the CX3CL1/CX3CR1-axis of the CX3C subfamily and TGF-beta2 were upregulated in SD-iCCA (Suppl. Fig. 1). Also, chemokine signaling pathways were downregulated in SD-iCCA, a.o. JAK2/3, PI3K, ERK1/2 and PKC (Suppl. Fig. 2).

## Discussion

By applying NanoString® technology, we exploratorily identified substantial differences in the local immune patterns of patient with SD- and LD-iCCA, including signatures of inflammation and immune response. By analyzing treatment-naïve tumor samples, our results suggest that the immune signatures are an intrinsic trait of the tumor types. Further, we corroborated our results by performing a multitude of complementary NanoString® technology analysis like pathway scoring, gene set enrichment analysis, differential expression analysis and cell type profiling to create a holistic model of the immune signatures in both iCCA subtypes. Considering the rapid emergence of immune-oncology diagnostics and treatment, our results provide insights and give evidence which can be used for biomarker discovery and to inform future studies with therapeutic intend before clinical translation.

Our data indicate that immune-related pathways are broadly downregulated in SD-iCCA, with the exception of complement signatures. Among 27 strongly differently expressed genes, 20 were downregulated in SD-iCCA with CEACAM6, DMBT1 and CD79A showing the strongest effect. From the 7 upregulated genes, CRP showed the highest differential expression and the complement factors C5, C4BPA, C8A and C8B comprised 57% of the upregulated genes. Remarkably, total TIL signatures were reduced in SD-iCCA. Cell type signatures differed in both iCCA subtypes and chemokine as well as cytokine-cytokine receptor interaction pathways were broadly downregulated in SD-iCCA.

In recent years, the nihilistic approach to the treatment of iCCA has been replaced by new therapies in the field of molecular and immunotherapeutic regimens. However, the heterogeneity of both iCCA subtypes and the lack of predictive biomarkers remain a major challenge in determining which patient subgroup will benefit from immunotherapy in order to provide stratified medicine. In addition to the known heterogeneity of SD- and LD-iCCA in terms of survival, response to chemotherapy, and molecular alterations, this study is the first to address differences in immune patterns. Our data demonstrate a downregulation of DMBT1 in SD-iCCA compared to LD-iCCA. DMBT1 is a mucin-like molecule that exerts functions in the regulation of epithelial differentiation and inflammation participating in mucosal immune defense (Mollenhauer et al. 2000; Bathum Nexoe et al. 2020). So far, only two studies investigated the role of DMBT1 in iCCA. Goeppert and colleagues did not observe differences in DMBT1 expression in iCCA compared to normal biliary tissue while they identified a significant decrease of DMBT1 expression in iCCA compared to biliary intraepithelial neoplasia (BilIN) 3 suggesting a tumor suppressing role of DMBT1 in early cholangiocarcinogenesis (Goeppert et al. 2017). In line, Sasaki et al. demonstrate a decreased expression of DMBT1 in tissue of invasive iCCA compared to intraductal papillary neoplasms (Sasaki et al. 2003). Against this background, one would rather suspect DMBT1 downregulation in LD-iCCA as this subtype is commonly associated with impaired survival and a more aggressive tumor biology (Kinzler et al. 2022). However, lack of DMBT1 expression in non-neoplastic biliary tissue of CCA patients was associated with poor survival while no significant impact on outcome was observed when DMBT1 expression was reduced in cancer cells (Goeppert et al. 2017). Interestingly, overexpression of DMBT1 was shown in primary sclerosing cholangitis as well as in hepatolithiasis (Bisgaard et al. 2002; Sasaki et al. 2003), two common risk factors for the occurrence of LD-iCCA (Aishima and Oda 2015). This may explain the upregulation of DMBT1 in LD-iCCA as hepatolithiasis was significantly more present in in this cohort in our study. It should be noted that both studies did not differentiate between iCCA subtypes, which may hinder comparability to our data. Further, we found a downregulation of CEACAM6 in SD-iCCA compared to LD-iCCA. CEACAM6 is a member of the immunoglobulin cell adhesion molecule superfamily, and its overexpression is associated with poor prognosis and invasiveness in vivo and in vitro in iCCA (Ieta et al. 2006; Liu et al. 2022; Kurlinkus et al. 2021). In addition, high levels of CEACAM6 are suggested as a screening parameter especially for eCCA (Rose et al. 2016), which is consistent with our data showing overexpression of CEACAM6 in LD-iCCA, as LD-iCCA are generally mucin-secreting tubular adenocarcinomas resembling perihilar and distal CCA (Kendall et al. 2019). Intriguingly, Ieta et al. could demonstrate that CEACAM6 overexpression is associated with chemoresistance to gemcitabine in vitro (Ieta et al. 2006) while two recently published studies revealed significant shorter progression-free survival for LD-iCCA receiving gemcitabine-based chemotherapy (Kinzler et al. 2022; Yoon et al. 2021). Thus, our data suggest that CEACAM6 could serve as a potential chemoresistant marker to gemcitabine especially in patients suffering from LD-iCCA.

Complement proteins, as a part of tumor microenvironment, can play a pivotal role in local immune response in various cancer entities (Roumenina et al. 2019). A recent proteomic analysis demonstrated that complement factors were significantly increased in CCA patients (Son et al. 2020) while it was shown that the reduced expression of complement factor H-related 3 is associated with poor prognosis and immune regulation in CCA patients (Wang et al. 2022). As such, presence of complement factor H-related 3 negatively correlated with tumor infiltrating lymphocytes like CD8 + T cells (Wang et al. 2022). In line with these findings, our data show an upregulation of various complement factors and a concomitant decrease of TIL in SD- compared to LD-iCCA. As part of the adaptive immune system, TIL can either target tumor cells to prevent carcinogenesis and cancer progression, or cancer cells can adopt strategies to evade the immune responses against the cancer, thus promoting tumor progression (Gooden et al. 2011). TIL comprise of highly heterogeneous immune cells, including CD8 + T cells. In iCCA, two studies have shown that an increase in TIL is associated with favorable outcome (Xia et al. 2022a; Yoon et al. 2021) while Goeppert et al. confirmed the prognostic value of TIL only for eCCA, but not for iCCA (Goeppert et al. 2013). For the first time, we could show increased CD8 + /TIL ratios in SD-iCCA compared to LD-iCCA in our study, which is in line with Xia et al. and Yoon et al. as this subtype is generally associated with better overall survival (Kinzler et al. 2022). However, Yoon et al. investigated TIL solely by determining the CD8 + status by immunohistochemistry in a sub cohort of PD-L1-inhibitor treated patients in recurrent or unresectable CCA that underwent upfront chemotherapy, which likely affected the immune landscape (Yoon et al. 2021), while we used NanoString® technology in treatment naïve tumor tissue. Thereby, our results were not potentially cofounded by prior treatments and thereby indicate a tumor-intrinsic trait.

In general, the presence of TIL- and chemokine-infiltrated TME is associated with better response to immune checkpoint blockade in CCA (Binnewies et al. 2018). So far, both iCCA subtypes are traditionally merged in the clinical context and are treated similarly with regard to chemo- and immunotherapeutic approaches. This long-held hypothesis is challenged by our finding that the immune signatures of LD-iCCA comprise higher levels of total TIL and chemokine signaling. Therefore, our results might suggest that this subtype is potentially more responsive to immunotherapy. However, studies investigating the potentially different response to immunotherapy in SD- and LD-iCCA with a possible link to TIL expression are needed.

Our study has several limitations that warrant discussion. We performed a retrospective analysis and selection bias cannot be ruled out. Our study population was small, and generalizability may not be presumed as this study was exploratory and hypothesis-generating in nature. However, we used treatment naïve tumor tissue of surgically resected specimen which ensured that the immune landscape of our samples was not altered due to prior anti-cancer treatments. Given these considerations, the substantial novelty of our data and the fact that data on this topic are lacking so far, the results of the present study are of high clinical relevance. Our aim was to exploratively analyze different immune patterns using NanoString® technology. We anticipate our results to accelerate and inform future work using confirmatory analysis such as immunofluorescence staining, flow cytometry, qPCR, in vitro, and in vivo experiments in prospective studies to augment and validate our results prior to clinical translation.

In conclusion, our study is the first to demonstrate that SD- and LD-iCCA are associated with dedicated local patterns of immune profiles. The substantial differences hold clinically relevant promise for biomarker discovery and treatment planning, especially using immune checkpoint therapy aimed at subtype-specific, personalized medicine. We anticipate our findings to inform future work which is needed to build upon and corroborate our findings, and thereby improve our understanding of iCCA biology for improved diagnostics, and treatment approaches.

### Supplementary Information

Below is the link to the electronic supplementary material.

Supplementary file1 (PDF 104 KB)

## Data Availability

The data that support the findings of this study are available from the corresponding author upon reasonable request.

## Abbreviations

BiIIN: Biliary intraepithelial neoplasia
CCA: Cholangiocarcinoma
CEACAM6: Carcinoembryonic antigen-related cellular adhesion molecule 6
DMBT1: Deleted in malignant brain tumor 1
FFPE: Formalin-fixed paraffin-embedded
HE: Hematoxylin and eosin
iCCA: Intrahepatic cholangiocarcinoma
LD-iCCA: Large duct type intrahepatic cholangiocarcinoma
UCT: University Cancer Center Frankfurt
UICC: Union for International Cancer Control
SD-iCCA: Small duct type intrahepatic cholangiocarcinoma
TIL: Tumor-infiltrating lymphocytes
TME: Tumor microenvironment
