# Supplementary material for: Small duct and large duct type intrahepatic cholangiocarcinoma reveal distinct patterns of immune signatures
Source: J Cancer Res Clin Oncol. 2024 Jul 22;150(7):357. doi: 10.1007/s00432-024-05888-y (PMC11271402; doi:10.1007/s00432-024-05888-y)
Supplement: Supplementary file 1 — Supplementary file1 (PDF 104 KB) [file 432_2024_5888_MOESM1_ESM.pdf]

Supplementary Files

Suppl. Fig. 1 *Pathview: Cytokine-Cytokine Receptor Interaction*

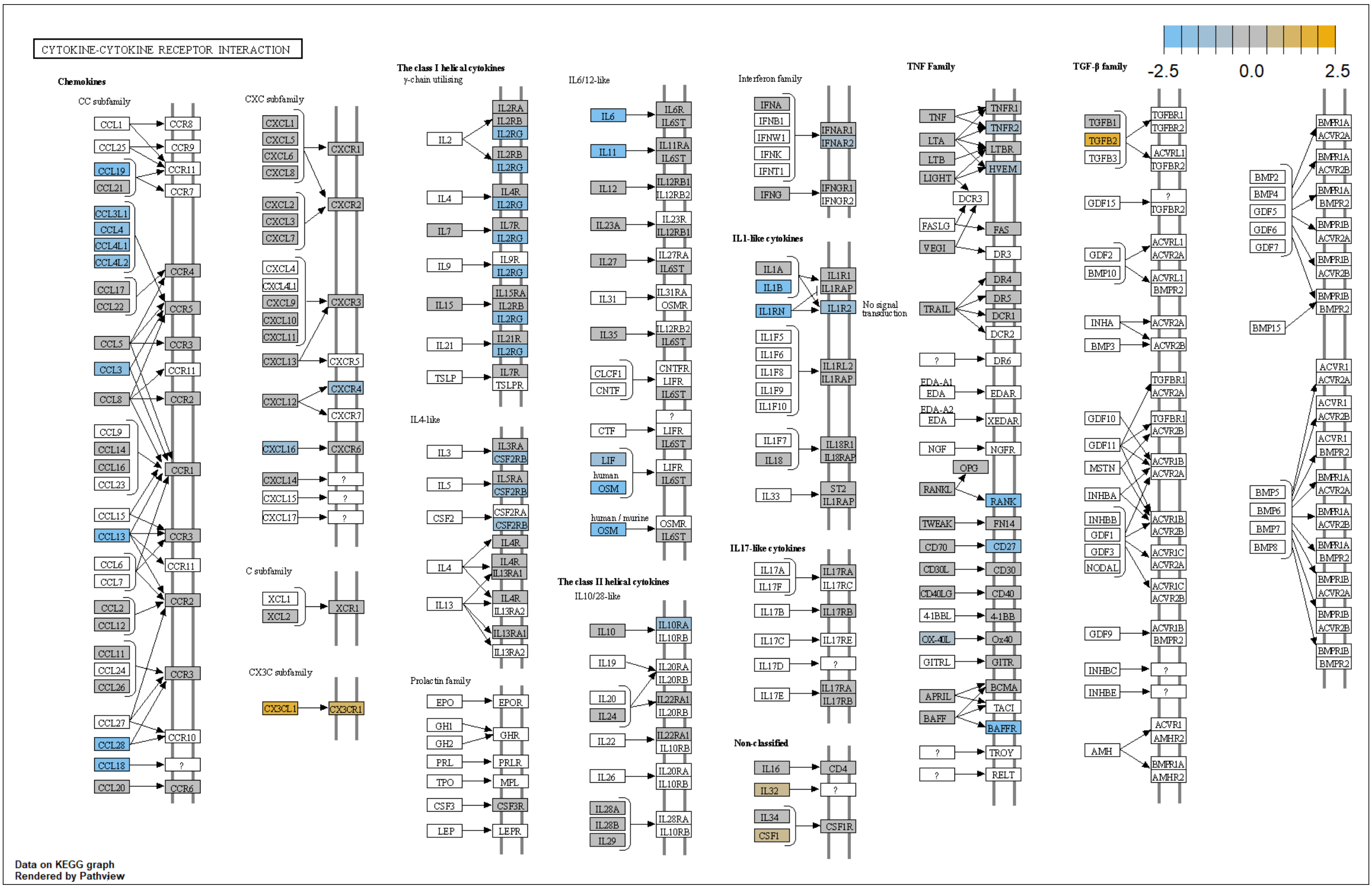

Pathview based on KEGG pathway with overlaid differential expression data. White nodes represent genes which were not represented in the panel. Grey nodes were not significantly differentially expressed. Blue nodes were downregulated and orange nodes were upregulated in SD-iCCA relative to our baseline of LD-iCCA.

Suppl. Fig. 2 *Pathview: Chemokine Signaling Pathway*

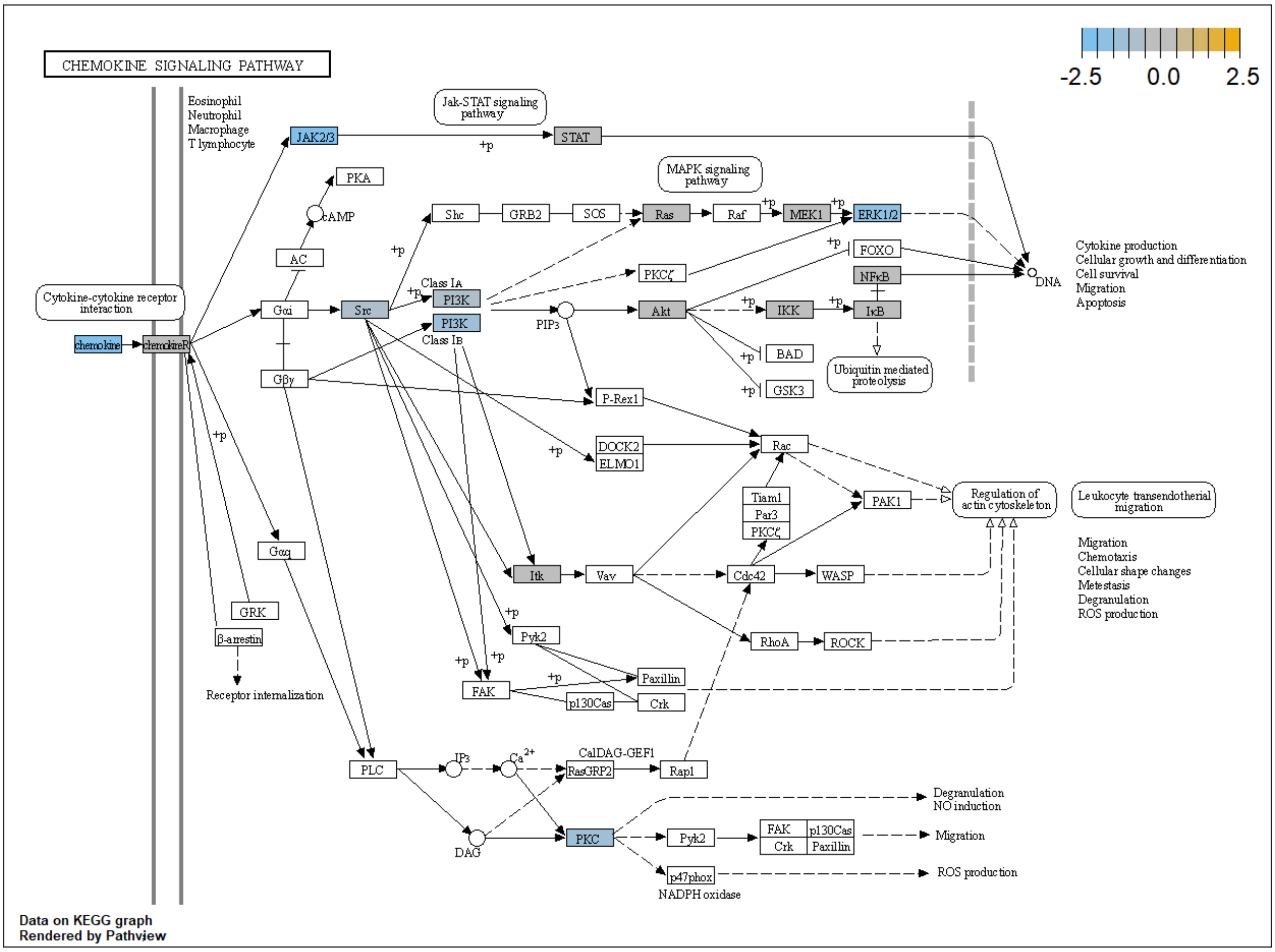

Pathview based on KEGG pathway with overlaid differential expression data. White nodes represent genes which were not represented in the panel. Grey nodes were not significantly differentially expressed. Blue nodes were downregulated and orange nodes were upregulated in SD-iCCA relative to our baseline of LD-iCCA.
